# PARP1 Differentially Interacts with Promoter region of *DUX4* Gene in FSHD Myoblasts

**DOI:** 10.4172/2157-7412.1000303

**Published:** 2016-08-08

**Authors:** Vishakha Sharma, Sachchida Nand Pandey, Hunain Khawaja, Kristy J Brown, Yetrib Hathout, Yi-Wen Chen

**Affiliations:** 1 Department of Molecular Medicine, George Washington University, Washington DC, USA; 2 Center for Genetic Medicine Research, Children's National Health System, Washington DC, USA; 3 Department of Integrative Systems Biology, George Washington University, Washington DC, USA

**Keywords:** Facioscapulohumeral muscular dystrophy, Myoblast, Immunoblotting, DUX4 Gene

## Abstract

**Objective:**

The goal of the study is to identity proteins, which interact with the promoter region of double homeobox protein 4 (DUX4) gene known to be causative for the autosomal dominant disorder Facioscapulohumeral Muscular Dystrophy (FSHD).

**Methods:**

We performed a DNA pull down assay coupled with mass spectrometry analysis to identify proteins that interact with a DUX4 promoter probe in Rhabdomyosarcomca (RD) cells. We selected the top ranked protein poly (ADP-ribose) polymerase 1 (PARP1) from our mass spectrometry data for further ChIP-qPCR validation using patients' myoblasts. We then treated FSHD myoblasts with PARP1 inhibitors to investigate the role of PARP1 in the FSHD myoblasts.

**Results:**

In our mass spectrometry analysis, PARP1 was found to be the top ranked protein interacting preferentially with the *DUX4* promoter probe in RD cells. We further validated this interaction by immunoblotting in RD cells (2-fold enrichment compared to proteins pulled down by a control probe, p<0.05) and ChIP-qPCR in patients' myoblasts (65-fold enrichment, p<0.01). Interestingly, the interaction was only observed in FSHD myoblasts but not in the control myoblasts. Upon further treatment of FSHD myoblasts with PARP1 inhibitors, we showed that treatment with a PARP1 inhibitor, 3-aminobenzamide (0.5 mM), for 24 h had a suppression of *DUX4* (2.6 fold, p<0.05) and *ZSCAN4, a* gene previously shown to be upregulated by *DUX4,* (1.6 fold, p<0.01) in FSHD myoblasts. Treatment with fisetin (0.5 mM), a polyphenol compound with PARP1 inhibitory property, for 24 h also suppressed the expression of *DUX4* (44.8 fold, p<0.01) and *ZSCAN4* (2.2 fold, p<0.05) in the FSHD myoblasts. We further showed that DNA methyltransferase 1 (DNMT1), a gene regulated by PARP1 was also enriched at the DUX4 promoter in RD cells through immunoblotting (2-fold, p<0.01) and immortalized FSHD myoblasts (42-fold, p<0.01) but not control myoblasts through ChIP qPCR.

**Conclusion:**

Our results showed that PARP1 and DNMT1 interacted with *DUX4* promoter and may be involved in modulating *DUX4* expression in FSHD.

## Background

Facioscapulohumeral muscular dystrophy (FSHD) is a digenic disorder and the third most common inherited form of muscular dystrophy [1,2]. The disease is characterized by a progressive and selective weakness and atrophy of the facial, scapular, and humeral muscles followed by weakness of muscles of the lower extremities. The weakness of muscles is often asymmetric [3]. There is currently no pharmacological therapy available to treat this disease.

While clinically indistinguishable, FSHD is sub classified into FSHD1 and FSHD2 based on the genetic causes [4-6]. FSHD1 is genetically linked to contractions of the D4Z4 repeat array at chromosome 4q35 and affects approximately 95% of patients. In patients with FSHD1, the D4Z4 array is contracted from 11-150 repeat units to 1-10 repeat units [4,5]. Individuals who do not have subtelomeric 4q35 region which contain the D4Z4 array do not develop FSHD [7]. Each of the repeat units contains a conserved ORF of the double homeobox 4 (*DUX4*) gene [8,9]. The FSHD permissive alleles contain a poly-adenylation signal in the pLAM region distal to the repeat array, which allows *DUX4* transcripts from the last D4Z4 repeat to be polyadenylated and therefore stabilized for protein translation [10,11]. FSHD2 is not linked to contractions of the D4Z4 repeat array. Recent studies have reported that some of the patients with FSHD2 have mutations in the structural maintenance of chromosomes flexible hinge domain containing 1 (*SMCHD1*) gene on chromosome 18 [12,13]. In addition, SMCHD1 was shown to be a genetic modifier in FSHD1 [13]. *SMCHD1* has been reported to play an important role in regulating DNA methylation by affecting chromatin structure [14-17]. The mutation of *SMCHD1* is believed to contribute to DNA hypomethylation of the D4Z4 region, and derepression of the *DUX4* gene.

The DUX4 protein is a homeodomain transcription factor that has been shown expressed in germ cells and causes upregulation of genes involved in gametogenesis in affected muscles when aberrantly expressed [18]. Previous studies showed that ectopic expression of DUX4 is cytotoxic both *in vitro* and *in vivo* [18-24]. Aberrant expression of *DUX4* in muscle cells has been reported to affect molecular pathways involved in myogenesis, oxidative stress responses, immune responses and germ line functions [18-20,23,25,26]. However, how the molecular and cellular changes cause the muscle pathologies and disease phenotypes is not clear.

Previous studies reported epigenetic changes of the D4Z4 region in FSHD, including loss of H3K9 trimethylation and HP1 gamma/cohesin binding, suggesting loss of heterochromatin. In addition, DNA hypomethylation of D4Z4 region in both FSHD1 and FSHD2 was reported, suggesting de-repression of gene expression in the region [27-35]. While the epigenetic mechanisms involved in de-repression of the *DUX4* have been studied extensively, the gene regulatory proteins that interact with the *DUX4* promoter and regulate the *DUX4* expression are not clear. In this study, we performed a DNA pull-down assay coupled with mass spectrometry to identify proteins that interacted with a *DUX4* promoter probe. We further validated interaction between the top ranked protein and the *DUX4* promoter using chromatin immunoprecipitation (ChIP). Last, we performed molecular assays using inhibitors against the protein to determine the functional outcome.

## Experimental Procedures

### Cell culture and nuclear extracts preparation

#### Cell culture and DNA pull-down assay

Rhabdomyosarcoma (RD) cells (ATCC) were cultured to 70% confluence in Dulbecco's modified Eagle's medium (Life Technologies) containing 10% heat inactivated fetal bovine serum (Sigma-Aldrich) and 1% penicillin-streptomycin (Life Technologies) at 37°C, 5% CO_2_. Retinal Pigment Epithelium (ARPE-19) cells (ATCC) were cultured to 70% confluence in Dulbecco's modified Eagle's medium and F-12 nutrient mixture supplemented with 1% penicillin streptomycin (Gibco) and 10% heat-inactivated fetal bovine serum (Sigma-Aldrich) at 37°C, 5% CO_2._ We used data generated from the ARPE-19 cells to further narrow down our final protein list to those more specific to muscle cells.

To prepare nuclear extracts, cells were first rinsed twice in 1× PBS (Life Technologies), then scraped with a cell scraper and finally collected in 50 ml conical tubes in PBS. The cell suspension was centrifuged at 1000 rpm for 5 min at 4°C and supernatant was removed. The cell pellet was then washed twice with 30 ml of 1× PBS and centrifuged at 1000 rpm and 4°C for 5 min. The packed cell volume (PCV) of pellet was estimated. Subsequently, 5× PCV volume of buffer A (10 mM HEPES (Acros Organics), 1.5 mM MgCl_2_ (Ambion), 10 mM KCl (Ambion), 2 mM DTT (Sigma-Aldrich), 200 μM PMSF (Sigma-Aldrich) and 1 tablet of protease inhibitor cocktail (PIC) (Roche Applied Sciences) was added to the pellet, mixed well by pipetting and incubated on ice for 10 min. Following incubation, the pellet was homogenized with a hand-held homogenizer. A small aliquot of cell pellet was then mixed with an equal volume of Trypan Blue to assay the extent of homogenization, which was always determined to be complete. The cell lysates were then centrifuged at 4°C for 10 min at 3000 rpm and the supernatant (cytoplasmic extract) was collected and frozen at -80°C. The pellet was centrifuged again at 14000 rpm for 15 min at 4oC and the remaining supernatant was discarded.The nuclear isolates in the pellets were subsequently extracted by adding two volumes of buffer C (20 mM HEPES, 25% glycerol, 0.42 M NaCl, 1.5 mM MgCl_2,_ 0.2 mM EDTA, 2mM DTT, 200 μM PMSF, 5 mM NaF and 1 PIC tablet) followed by homogenization and incubation on ice for 30 min. The lysates were centrifuged for 10 min at 4°C and 14000 rpm. Clear supernatant (nuclear extract) was subsequently collected and aliquoted for use in the DNA pull-down assays.

The DNA pull-down assay was performed based on a previously described procedure [36]. Briefly, the *DUX4* and *HTRA1* probes were amplified from human genomic DNA by the Polymerase Chain Reaction (PCR) in the 2720 Thermal Cycler (Applied Biosystems) with GoTaq Green Master Mix (Promega) at the following conditions: 94°C for 5 min and 40 cycles of 94°C for 45 s, 58°C for 30 s, 72°C for 90 s and 72°C for 10 min. The promoter probe of *HTRA1* gene is used as control. The following biotin-labeled primers were used in the PCR reaction: *DUX4* (forward): 5′-GGGCTGTCCCAGG-3′ and (reverse): 5′-GTCTCCGTCGCCG-3′; *HTRA1* (forward) 5′-GAATACG-GACACGCAT-3′ and (reverse): 5′- GCCCCTGCAGTCC-3′. The PCR products were subsequently purified through the QIAGEN Gel Extraction Kit. In the immunoblotting experiment, a random control probe (5′-AGAGTGGTCACTACCCCCTCTG-3′) was used.

Nuclear extracts (0.7 mg) from RD cells were incubated with the *DUX4* probes, while nuclear extracts from RPE cells were incubated with *HTRA1* probes (1.0 μg) in 1 ml binding buffer for 1 h at room temperature. Following the protein-probe binding, samples were incubated with 100 μl of the beads and mixed on a rotating arm at 4°C overnight. The beads were then centrifuged and washed twice in 500 μl of binding buffer with centrifugation performed between each wash step. The beads were then resuspended in 1× LDS sample buffer (Life technologies) and 1× reducing agent (Life technologies) and heated at 70°C for 10 min for SDS-PAGE electrophoresis.

#### Protein fractionation and in-gel digestion

Proteins pulled down from control and experimental samples were further separated by electrophoresis on a 4-12% Bis-Tris gel (Life Technologies) for 1 h at 100 V. The gel was stained with Coomassie Blue (Bio-Rad) to visualize proteins bands. Whole lanes were then sliced out in 2 mm pieces and processed for in-gel digestion, as described hereafter. Coomassie Blue and SDS were first removed from the gel pieces through sequential washes with 200 μl of water once, and 200 μl of 50% acetonitrile (Sigma-Aldrich) (15 min each) twice. Following removal of liquids, the gel pieces were shrunk immediately with 100 μl of 100% acetonitrile (Sigma-Aldrich) for 5 min. The gel pieces were then rehydrated with 100 μl of 100 mM NH_4_HCO_3_ (Sigma-Aldrich) for 5 min. An equal volume of 100% of acetonitrile (Sigma-Aldrich) was added and the gel pieces were incubated in the solution for another 15 min. All liquids were then removed and replaced with 100 μl of 100% acetonitrile (Sigma-Aldrich) until the gel pieces turned white. In-gel digestion was performed by rehydrating dried gel pieces in 20 μl of digestion buffer, consisting of 12.5 ng/μl of trypsin (Promega) in 50 mM of NH_4_HCO_3_ (Sigma-Aldrich) for 45 min in an ice bath. Excess digestion buffer was then removed and replaced with 5 μl of 50 mM NH_4_HCO_3_ and incubated overnight at 37°C. The peptides were extracted from the digested gel pieces by incubating the gel pieces with 25 μl of 25 mM NH_4_HCO_3_ for 15 min, with occasional vortexing. Equal volumes of 100% acetonitrile (Sigma-Aldrich) were added to each tube for another 15 min, with occasional vortexing. The supernatant was collected and the gel pieces were subjected to two additional extractions with 30 μl of 5% formic acid and 5% of formic acid-acetonitrile (1:1, v/v) for 10 min. The samples were vortexed twice during the incubations. The supernatants from each gel piece were pooled together and dried completely in a vacuum centrifuge. The dried samples were subsequently subjected to Liquid Chromatography-Tandem Mass Spectrometry analysis as previously described [37].

Proteins pulled down by the *DUX4* probes were first compared to proteins pulled down only by agarose beads to filter out non-specific interactions. The proteins preferentially interacting with the *DUX4* probe were further filtered for nuclear proteins with DNA binding potential. Uniprot Protein Knowledgebase was used to determine the subcellular localization and functions of proteins. Afterwards, results from an independent study were further filtered to limit the list to changes specific to the RD cells in order to retain muscle specific interactions.

#### Immunoblotting

The proteins pulled down by DNA pull-down assays were separated by electrophoresis on 4-20% SDS-PAGE gradient gels at 100 V for 1 h. They were then transferred onto nitrocellulose at 100 V for 2 h at 4°C (Life Technologies). The blots were blocked for 1 h in 5% milk (Bio-Rad) prepared in PBS (Life Technologies), 0.1% Tween (Bio-Rad). They were subsequently incubated with anti-PARP1 antibody (Santa Cruz) or anti-DNMT1 antibody (Abcam) at 4°C overnight. The blots were then washed three times in 0.1% Tween in PBS (Sigma-Aldrich); 5% milk in PBS, and 0.1% Tween in PBS (Life Technologies) for 15 min each, respectively. The blots were then incubated with anti-rabbit HRP (GE Health Sciences) for 2 h at room temperature and then washed in 0.1% Tween in PBS three times for 15 min each. HRP was detected with ECL (Pierce) and chemiluminescence was detected with ECL films (Amersham Pharmacia Biosciences).

### Cell culture and chromatin immunoprecipitation assay

Immortalized human myoblasts were obtained from the Senator Paul Wellstone Muscular Dystrophy Cooperative Research Center at Boston Biomedical Research Institute [38]. The patient myoblast cell line was derived from the biceps of a 42 year old patient with mild muscle weakness (WS157). The control myoblasts were derived from the patient's 46 year old sibling without FSHD (WS161) [38]. These cells were cultured as previously described [25,37,39]. Briefly, proliferating myoblasts were cultured in a growth medium described previously in detail at 37°C, 5% CO2 [25]. The culture dish was coated with 0.1% gelatin (Sigma-Aldrich). The medium was changed to growth medium comprising of 15% Knock-Out Serum Replacement (Life Technologies) (KOSR) instead of fetal bovine serum after 72 h of culturing cells. After another 72 h, the cells were harvested and nuclear extracts were prepared. The KOSR was used because it has been shown to increase DUX4 expression [40].

Chromatin was first crosslinked to proteins in 10^6^ human immortalized cells by adding 1% formaldehyde to the cell culture medium. Nuclear extracts were prepared as described previously and chromatin was sonicated to generate fragment sizes of 200-1000 bp using five pulses of 10 s each (10% duty cycle). Sonication was performed using the Sonifier cell disruptor 350 (Branson Sonic Power, Smith Kline Co.) using 3 mm Branson converter. Afterward, the sonicated nuclear lysates were centrifuged. The supernatants were collected and diluted 10-fold in ChIP dilution buffer (EMD Millipore Chemicals) with protease inhibitor cocktail added to the buffer (Roche Applied Sciences). The diluted nuclear lysate was precleared with 75 μl of Salmon Sperm DNA/ Protein A Agarose-50% Slurry (EMD Millipore Chemicals) for 30 min at 4°C with agitation and subsequently pelleted by brief centrifugation. The samples were then incubated with 5 μg of rabbit polyclonal anti-PARP1 antibody (Santa Cruz) or DNMT1 antibody at 4°C on a shaker. Rabbit IgG (Abcam) was used as control (5 μg). Samples were then incubated with Salmon Sperm DNA/Protein A Agarose-50% Slurry for 1 h at 4°C with gentle agitation on a shaker to collect the antibody/protein complexes, which were subsequently pelleted with brief centrifugation. The pellets were sequentially washed with Low Salt Immune Complex Wash Buffer (EMD Millipore Chemicals), High Salt Immune Complex Wash Buffer (EMD Millipore Chemicals), LiCl Immune Complex Wash Buffer (EMD Millipore Chemicals), and TE Buffer (EMD Millipore Chemicals) for 5 min each at room temperature followed by one wash with TE Buffer at 4°C with agitation, and pelleted by centrifugation each time. The DNA was eluted by resuspending the pellets twice in 250 ul of 1% SDS and 0.1 M NaHCO_3_ for 15 min with brief centrifugation at the end of each incubation and combining both eluates. Cross-links were reversed by adding 20 μl of 5 M NaCl (Promega) to the combined eluates (500 μl) and incubating for 4 h at 65°C. Ten, 10 μl of 0.5 M EDTA (Ambion), 20 μl 1 M Tris-HCl, pH 6.5 (EMD Millipore Chemicals), and 2 μl of 10 mg/ ml Proteinase K (Ambion) were added to samples and incubated for 1 h at 45°C. DNA was extracted from the samples using phenol-chloroform extraction and resuspended in 10 μl of water. DNA was amplified in triplicates in SYBR Green PCR Master Mix (Life Technologies) using 1 μM of forward and reverse primers specific to the promoter fragments of each gene and 1 μl of the DNA template in a total volume of 50 μl. The thermal cycling conditions were 50°C for 2 min, 95°C for 10 min, followed by 40 cycles of amplification using the condition of 95°C for 15 s then 60°C for 1 min. The primers used to amplify the *DUX4* promoter fragments were (forward) 5′-ATTCATGAAGGGGTGGAGCC-3′ and (reverse) 5′- TGCACCTCAGCCGGAC-3′.

### Cell culture and PARP inhibitor study

A total of 9000 FSHD myoblasts were seeded and cultured in regular media in 25 cm^2^ flasks according to the protocol described in the previous section. The media was replaced with KOSR containing media 72 h after seeding the cells. The cells were treated with 0.5 mM of 3-AB and fisetin dissolved in DMSO 48 h after changing media for 24 h and subsequently harvested for RNA isolation. RNA isolation, cDNA synthesis, semi-quantitative RT-PCR for DUX4 and real time qRT-PCR for ZSCAN4 was performed as previously described [25]. Briefty, total RNA was isolated using mirVana kit (Ambion) according to the manufacturer's protocol. The total RNA thus isolated from (3 μg) from each sample was first subjected to DNAse I digestion (1 U) to remove genomic DNA contamination as previously described [25]. The RNA was then purified using the RNeasy MinElute Cleanup Kit (Qiagen) according to the manufacturer's protocol. Subsequently, the RNA sample was reverse transcribed to cDNA using Superscript III (Life Technologies) and oligo dT primers. The cDNA thus generated was amplified using GoTaq green master Mix (Promega) using 1 μM of forward and reverse primers specific to each gene and 3 μl of cDNA template in a total volume of 20 μl. The thermal cycling conditions included 95°C for 3 min, followed by 40 cycles of amplification using the condition of 95°C for 10 s then 62°C for 45 s. Ten further kept for 72°C for 10 min. Primer sequences used for human *DUX4* were (forward) 5′-CCCAGCTACCAGCAGACC-3′ and (reverse) 5′ TCCAGGAGATGTAACTCTAATCCA-3′. PCR products were taken from successive PCR cycles and resolved by electrophoresis in 2.0% agarose gel, stained with Ethidium Bromide (EtBr) and visualized under ultraviolet light and imaged using Ingenius Imaging System (Syngene). The samples which were analyzed and compared to each other (internal control, GAPDH and PARP1 inhibitors treated samples) were loaded on the same gel following same settings of the image analyses. Densitometric analysis of EtBr-stained gel bands was performed using Image J software (NIH). To analyze the human *zinc finger and SCAN domain containing 4* (*ZSCAN4*) we amplified the cDNA in triplicates in SYBR Green PCR Master Mix (Life Technologies) using 1 μM of forward and reverse primers specific to each gene and 1 μl of cDNA template in a total volume of 20 μl. The thermal cycling conditions included 50°C for 2 min, 95°C for 10 min, followed by 40 cycles of amplification using the condition of 95°C for 15 s then 60°C for 1 min. Primer sequences used for human *zinc finger and SCAN domain containing 4* (*ZSCAN4*) were (forward) 5′-TGGAAATCAAGTGGCAAAAA-3′ and (reverse) 5′-CTGCATGTGGACGTGGAC-3′ [24]. *Glyceraldehyde-3-phosphate dehydrogenase* (*GAPDH*) was used as internal control and the primers used were (forward) 5′- TGTCAAGCTCATTTCCTGGTA-3′ and (reverse) 5′-GTGAGGGTCTCTCTCTTCCTCTTGT-3′. Standard Ct method was used to calculate expression values relative to GAPDH among the samples (http://www3.appliedbiosystems.com/cms/groups/mcb_support/documents/generaldocuments/cms_042380.pdf).

## Results

### PARP1 interacts with the promoter of *DUX4* in immortalized FSHD myoblasts

**Figure 1:**
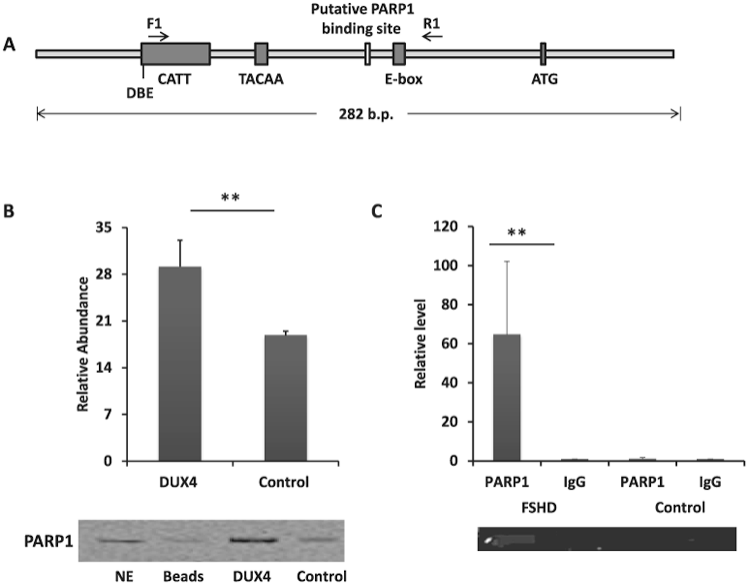
PARP1 interacts with the *DUX4* promoter in FSHD myoblasts. (A) Known and putative genomic and regulatory sequences within the *DUX4* promoter probe. The regulatory elements are the CATT box, TACAA box, DBE (D4Z4 binding element), putative PARP1 (Poly[ADP-ribose] polymerase 1) binding site, E box, and ATG (start codon). (B) Immunoblotting was performed using proteins pulled down with the *DUX4* promoter probe from nuclear extracts of RD cells. Proteins pulled down with a random probe served as the control for the study. The western blot is representative of four experiments and the densitometry figure shows mean ± SE. *p<0.05 (t-test). (C) ChIP for *DUX4* promoter was performed on immortalized FSHD myoblasts and control myoblasts of an unaffected sibling using PARP1 and IgG (control) antibodies (n=3). PARP1 enrichment at the *DUX4* promoter in the FSHD myoblasts was calculated by normalizing to the IgG samples. There was no enrichment of PARP1 at the *DUX4* promoter in the control myoblasts. The bars represent mean of relative fold-change ± SE. **p<0.01 (t-test).

To identify proteins that interact with the *DUX4* promoter, we performed a DNA pull-down assay coupled with mass spectrometry in RD cells. A 282 bp double stranded DNA probe encompassing 213 bp of the promoter region and 69 bp of the coding region of the *DUX4* gene was used (Figure 1A). The selected region contains several DNA regulatory elements including the CATT box, GC box, TACAA box and E b ox reported previously [10]. Additional potential regulatory elements that are outside this region were not examined in this study. Proteins pulled down by the *DUX4* probe were identified by mass spectrometry. The proteins were compared to proteins pulled down by beads only to remove non-specific interactions. We then further narrowed down the protein list by removing proteins pulled down by a probe designed against a non-muscle gene HtrA serine peptidase 1 (*HTRA1*) and retaining only nuclear proteins. A total of 12 proteins (Table 1) were identified with poly (ADP-ribose) polymerase 1 (PARP1) ranked at the top (Table1). No protein that has been reported to interact with the CATT box, GC box, TACAA box, or the E box was identified.

To validate the interaction between the PARP1 and the *DUX4* promoter probe, we repeated the DNA pull-down assays using RD cells, followed by immunoblotting assay. Our results demonstrated a 2-fold (p<0.05) enrichment of the PARP1 protein interacting with the *DUX4* promoter probe as compared with proteins pulled-down by a control probe with random sequences (Figure 1B).

To examine the interaction between PARP1 and *DUX4* promoter in human myoblasts, we performed a ChIP assay targeting the promoter region containing a putative PARP1 binding site located at 112-116 bp upstream of the transcription start site [41] using immortalized human myoblasts from an individual with FSHD and myoblasts from the subject's unaffected sibling as control [37]. A 123 bp amplicon in the *DUX4* promoter encompassing the PARP1 binding sites in the middle of the amplicon was designed for ChIP validation as shown in Figure 1A. In the immortalized FSHD myoblasts, real-time quantitative PCR analysis showed a 65-fold enrichment (p<0.01) of the *DUX4* promoter fragment in the DNA pulled down by PARP1 antibody as compared with the DNA pulled down by IgG. Interestingly, no significant enrichment of PARP1 was observed in the control myoblasts (Figure 1C).

### PARP1 inhibition leads to suppression of DUX4 and ZSCAN4 expression

**Figure 2:**
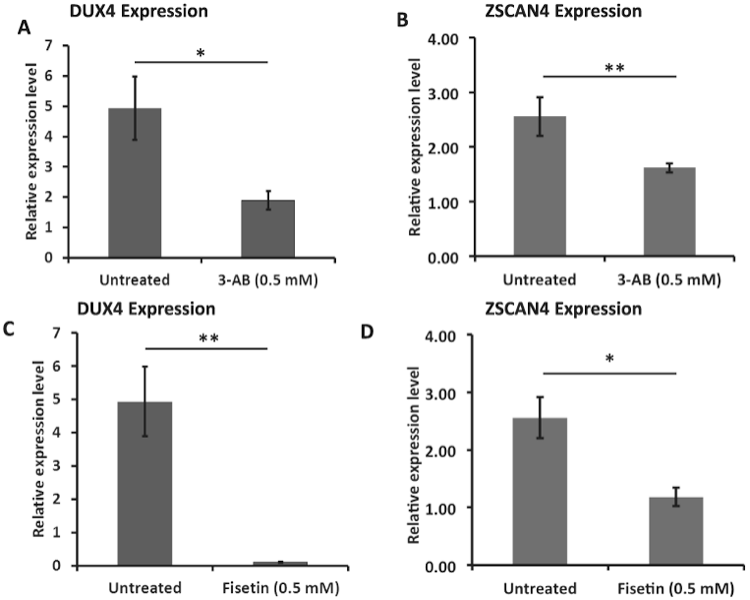
*DUX4* and *ZSCAN4* are downregulated in response to PARP1 inhibitors, 3-ABand fisetin, in immortalized FSHD myoblasts. Real-time quantitative PCR was performed on FSHD myoblasts treated with 0.5 mM 3 aminobenzamide (A and B) or fisetin (C and D) with untreated cells serving as the control (n=4). Relative expression level of *DUX4* (A and C) and *ZSCAN4* (B and D) were calculated relative to *GAPDH*. The bars represent mean of relative expression level ± SE. *p<0.05, **p<0.01 (t-test).

To determine functional significance of the interaction between the PARP1 and the *DUX4* promoter, we treated FSHD immortalized myoblasts with a commonly used PARP1 inhibitor, 3-aminobenzamide (3-AB). 3-AB mimics the substrate of PARP1, NAD+ and binds PARP1, thereby preventing PARP1 from utilizing NAD+ to poly(ADP-ribosyl) ate (PARylate) targets, which is necessary for PARP1 activity [42,43]. RT-PCR analysis showed 2.6 fold (p<0.05) suppression of *DUX4* expression after the immortalized FSHD myoblasts were treated with 0.5 mM 3-AB for 24 h (Figure 2A). We then examined expression of zinc finger and SCAN domain containing 4 (*ZSCAN4*), one of the germ line genes reported to be dramatically up-regulated by DUX4 previously [18,25,44]. Our results showed a suppression (1.6 fold, p<0.01) of *ZSCAN4* expression in response to 3-AB treatment in the FSHD myoblasts (Figure 2B).

Fisetin is a natural compound that was shown to significantly inhibit PARP1 [45]. We examined whether treatment of fisetin will also suppress DUX4 and ZSCAN4 expression in the FSHD myoblasts. RT-PCR analysis showed 44.8 fold (p<0.01) suppression of *DUX4* expression after the immortalized FSHD myoblasts were treated with 0.5 mM fisetin for 24 h (Figure 2C). Further, real-time quantitative PCR analysis showed suppression (2.2 fold, p<0.05) of *ZSCAN4* expression in response to fisetin treatment (Figure 2D). The data showed that the PARP1 inhibitors, 3-AB and fisetin, suppressed the expression of DUX4 and its downstream target, ZSCAN4.

### DNMT1 interacts with the promoter of *DUX4*

**Figure 3:**
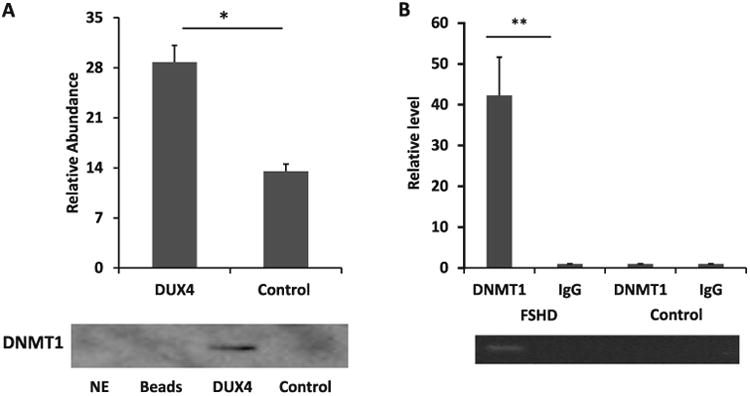
DNMT1 interacts with the *DUX4* promoter in FSHD myoblasts. (A) Immunoblotting was performed using proteins pulled down with the *DUX4* promoter probe from nuclear extracts of RD cells. Proteins pulled down with a random probe served as the control for the study. The western blot is representative of four experiments and the densitometry figure shows mean ± SE. *p<0.05 (t-test). (B) ChIP for *DUX4* promoter was performed on immortalized FSHD myoblasts and control myoblasts of an unaffected sibling using DNMT1 and IgG (control) antibodies (n=3). DNMT1 enrichment at the *DUX4* promoter in the FSHD myoblasts was calculated by normalizing to the IgG samples. There was no enrichment of DNMT1 at the *DUX4* promoter in the control myoblasts. The bars represent mean of relative fold-change ± SE. **p<0.01 (t-test).

PARP1 has been shown to modulate gene expression by inhibiting the catalytic activity of DNMT1, which leads to DNA de-methylation. Considering that this function involves PARP1 forming a complex with DNMT1 at the promoter of a target gene [46,47], we examined whether DNMT1 interacts with the *DUX4* promoter in RD cells using DNA pull-down assay coupled with immunoblotting. The result showed that the DNMT1 proteins were pulled down by the *DUX4* promoter probe (2 fold, p<0.01) (Figure 3A).

We further examine the interaction of DNMT1 with the promoter in the immortalized myoblasts. The same 123 bp amplicon in the *DUX4* promoter encompassing the PARP1 binding sites in the middle of the amplicon was designed for ChIP validation as shown in Figure 1A. In the immortalized FSHD myoblasts, real-time quantitative PCR analysis showed a 42-fold enrichment (p<0.01) of the *DUX4* promoter fragment in the DNA pulled down by DNMT1 antibody as compared with the DNA pulled down by IgG. Interestingly, no significant enrichment of DNMT1 was observed in the control myoblasts (Figure 3B).

## Discussion

In this study, the DNA pull-down assay in combination with mass spectrometry analysis was implemented to identify novel gene regulators of *DUX4* gene. We used highly stringent criteria to narrow down the identified proteins to 12 proteins. The highest ranked PARP1 was further validated by DNA pull-down assay using the same DNA promoter probe, followed by immunoblotting. We further showed that the interaction between PARP1 and the DUX4 promoter was only detected in FSHD myoblasts but not control myoblasts, suggesting that the DUX4 promoter in the control myoblasts may be inaccessible to the PARP1. This may be due to structural differences of chromatin, which has been shown to be altered in FSHD myoblasts [48-50]. Studies have shown that the D4Z4 region in FSHD patients are associated with loss of the heterochromatin marks H3K9 trimethylation as well as disrupted HP1 gamma/cohesin binding [32,33]. The D4Z4 region has also been shown to be hypomethylated in patients [27-29,34,35]. These altered chromatin patterns on the D4Z4 region in patients may allow interaction between PARP1 and *DUX4* promoter in patients' myoblasts.

PARP1 is a chromatin-associated protein involved in the post-translational modification of various nuclear proteins. It functions in various cellular processes such as genomic maintenance, chromatin structure, transcription, replication, cell cycle regulation and cell death through several mechanisms including post-translational modification of other proteins with poly (ADP-ribose) chains (PARs), direct interactions with other proteins, and DNA binding [48-60]. PARP1 has been shown to loosen chromatin by modifying proteins attached to chromatin through poly-ADP-ribosylation (PARylation), thereby leading to the formation of localized puffs that permit transcription [61]. In addition, previous studies showed that PARP1 interacted with promoters and affected the gene expression by replacing other nuclear proteins that maintain chromatin structures [62,63]. A recent study suggested that the loss of H3K9 trimethylation pattern may displace SMCHD1 from the D4Z4 region and contribute to increased *DUX4* expression [33]. Since we only observed the interaction between the PARP1 and the DUX4 promoter in the FSHD cells but not the control cells, we believe that the shortening of the D4Z4 array created a genomic environment that is more accessible to PARP1.

PARP1 has also been shown to regulate methylation patterns by regulating DNMT1 expression. PARP1 can directly activate *DNMT1* transcription as well as regulate its enzymatic function via PARylation [64]. PARP1 was reported to interact and co-localize with DNMT1 at the promoter of target genes. ADP-ribose polymers were added to the DNMT1 by PARP1, which suppresses its ability of methylating the target DNA sequence [42,48,65,66]. DNA de-methylation then leads to activation of the target genes. While patients with FSHD1 and FSHD2 have different primary genetic defects, some epigenetic features are shared in both groups of patients, including changes of histone marks indicating a more open chromatin structure and DNA hypomethylation suggesting active transcription activities in the D4Z4 region [27,28,32,34,35,67]. Our results showed that PARP1 and DNMT1 interacted with the *DUX4* promoter, suggesting that this interaction may potentially play a significant role in the demethylation and subsequent upregulation of *DUX4* observed in patients with FSHD.

PARP1 has been shown to facilitate transcription activation of NFkB by acting as a co-activator [68]. It has also been shown increased in inflammatory disorders through NFkB-dependent pathways [69]. FSHD has been shown to exhibit inflammatory features in the form of upregulation of both adaptive and innate immune response and the FSHD myoblasts have been shown to be more susceptible to oxidative stress [70-72]. Several PARP1 inhibitors have been shown effective in treating inflammatory disorders. Both PARP inhibitor 3-AB and fisetin have been shown to have anti-inflammatory properties [73-75]. In addition, both agents have also been studied for their anti-oxidative property [76,77]. Considering the function of PARP1 inhibitors and the molecular defects of FSHD, agents with PARP1 inhibiting properties can be investigated further for potential therapeutic applications.

In this study, we identified that PARP1 and DNMT1 interact with the *DUX4* promoter through immunoblotting. The interactions in FSHD myoblasts were further validated by ChIP. We were unable to detect DNMT1 interaction with the *DUX4* promoter through mass spectrometry likely because mass spectrometry is sometimes not sensitive enough to detect presence of proteins that are not very abundant. Immunoblotting has a higher sensitivity of detecting proteins than mass spectrometry, which possibly explains why DNMT1 interaction with *DUX4* promoter was detected by immunoblotting but not mass spectrometry. The interaction was then further validated by ChIP. The interaction between PARP1 and DNMT1 and the promoter was further demonstrated in FSHD myoblasts but not the control cells through ChIP assay. It should be noted that there is no difference of PARP1 expression between control and FSHD myoblasts based on previously published profiling studies [10,26,78]. In order to increase DUX4 expression in immortalized FSHD myoblasts during ChIP assay, we cultured these cells in growth medium containing 15% KOSR instead of 15% FBS. We have recently shown that while *DUX4* expression was difficult to be detected in proliferating myoblasts cultured with 15% FBS, it was readily detected in myoblasts cultured with 15% KOSR [79]. This culture condition allows us to study factors that are regulating DUX4 expression.

Treatments of PARP1 inhibitors 3-AB and fisetin suppressed the expression of *DUX4* and its downstream target, *ZSCAN4*. Based on previous findings and data from this study, we hypothesized that the alteration of the chromatin due to the contraction of D4Z4 array allows PARP1 to interact with the *DUX4* promoter. This may either facilitate the transcription of DUX4 via interacting with other transcription factors or contribute to the DNA hypomethylation in the region by inhibiting DNMT1, which has been proposed to lead to the de-repression of the *DUX4* gene. In addition, our study identifies two PARP1 inhibitors, namely 3-AB and fisetin, as novel suppressors of *DUX4* expression, and plausible candidates for therapeutic development for FSHD.

## Figures and Tables

**Table 1:** Proteins interacting preferentially with the *DUX4* promoter probe.

Protein symbols	Protein names	Peptide Hits
**PARP1**	Poly (ADP-ribose) polymerase 1	8
**MSH2**	DNA mismatch repair protein	4
**MCM2**	DNA replication licensing factor	3
**SFRS5**	Serine/arginine-rich splicing factor 5	2
**IF4A3**	Eukaryotic initiation factor 4A-III	1
**HNRDL**	Heterogeneous nuclear ribonucleoprotein D-like	1
**IF2B1**	Insulin-like growth factor 2 mRNAbinding protein 1	1
**PABP2**	Polyadenylate-binding protein 2	1
**TADBP**	TAR DNA-binding protein 43	1
**KC1D**	Casein kinase I isoform delta SWI/SNF-related matrix-associated	1
**SNF5**	actin-dependent regulator of chromatin subfamily B member 1	1
**COPB**	Coatomer subunit beta	1

## Abbreviations

FSHD: Facioscapulohumeral Muscular Dystrophy
DUX4: Double Homeobox 4
RD: Rhabdomyosarcoma
ChIP: Chromatin Immunoprecipitation Assay
qPCR: Quantitative Polymerase Chain Reaction
PARP1: Poly (ADP-ribose) Polymerase 1
3-AB: 3-Aminobenzamide
ZSCAN4: Zinc finger and SCAN domain containing 4
KOSR: Knockout Serum Replacement
